# Protein Array-based Approaches for Biomarker Discovery in Cancer

**DOI:** 10.1016/j.gpb.2017.03.001

**Published:** 2017-04-07

**Authors:** Yi Huang, Heng Zhu

**Affiliations:** 1 Provincial Clinical College, Fujian Medical University, Fuzhou 350001, China; 2 Department of Pharmacology & Molecular Sciences, Johns Hopkins University School of Medicine, Baltimore, MD 21205, USA

**Keywords:** Protein array, Biomarker, Cancer, Proteomics, Early diagnosis

## Abstract

**Biomarkers** are deemed to be potential tools in **early diagnosis**, therapeutic monitoring, and prognosis evaluation for **cancer**, with simplicity as well as economic advantages compared with computed tomography and biopsy. However, most of the current cancer biomarkers present insufficient sensitivity as well as specificity. Therefore, there is urgent requirement for the discovery of biomarkers for cancer. As one of the most exciting emerging technologies, **protein array** provides a versatile and robust platform in cancer **proteomics** research because it shows tremendous advantages of miniaturized features, high throughput, and sensitive detections in last decades. Here, we will present a relatively complete picture on the characteristics and advance of different types of protein arrays in application for biomarker discovery in cancer, and give the future perspectives in this area of research.

## Introduction

Biological markers (biomarkers) are defined as “cellular, biochemical or molecular alterations that are measurable in biological media such as human tissues, cells, or fluids” [1]. Such alterations can be caused by various factors, such as germline or somatic mutations, transcriptional changes, and posttranslational modifications (PTMs). To this date, a wide variety of biomarkers in the forms of proteins (*e.g.*, antigens or antibodies), nucleic acids (*e.g.*, mutations in genomic DNA, microRNAs, or other non-coding RNAs), or protein PTMs, have been identified and routinely used for clinical diagnoses of different diseases including cancers [2].

In recent years, numerous technologies have been applied for discovery of biomarkers to improve early diagnosis, therapeutic stratification, and prognosis for cancer patients who have undergone treatments. Protein array/microarray is one of the most exciting emerging technologies, which is deemed to serve as a versatile and robust tool in cancer proteomics research due to its tremendous advantages of miniaturized features, high throughput, and sensitive detections [3], [4], [5]. When compared with the DNA array technology in particular, the protein array-based approaches are capable of analyzing a wide variety of biochemical properties of the protein entities, the ultimate driving force in a cell [6]. Fabricated by arraying with hundreds to thousands of individually-purified proteins (or mixtures of proteins) at extremely high density on a solid surface, the protein array technology allows for simultaneous investigations of hundreds of thousands of targets in a single experiment. Moreover, various recent advances in the array technologies have demonstrated that this platform is suitable for the discovery of novel biomarkers [6]. Here, we summarize different protein array platform-based technologies and their specific applications in the cancer biomarker research, which is expected to demonstrate the potential and promote the use of this relatively new technology in the field of translational medicine.

## Overview of the protein array technology

**Figure 1:**
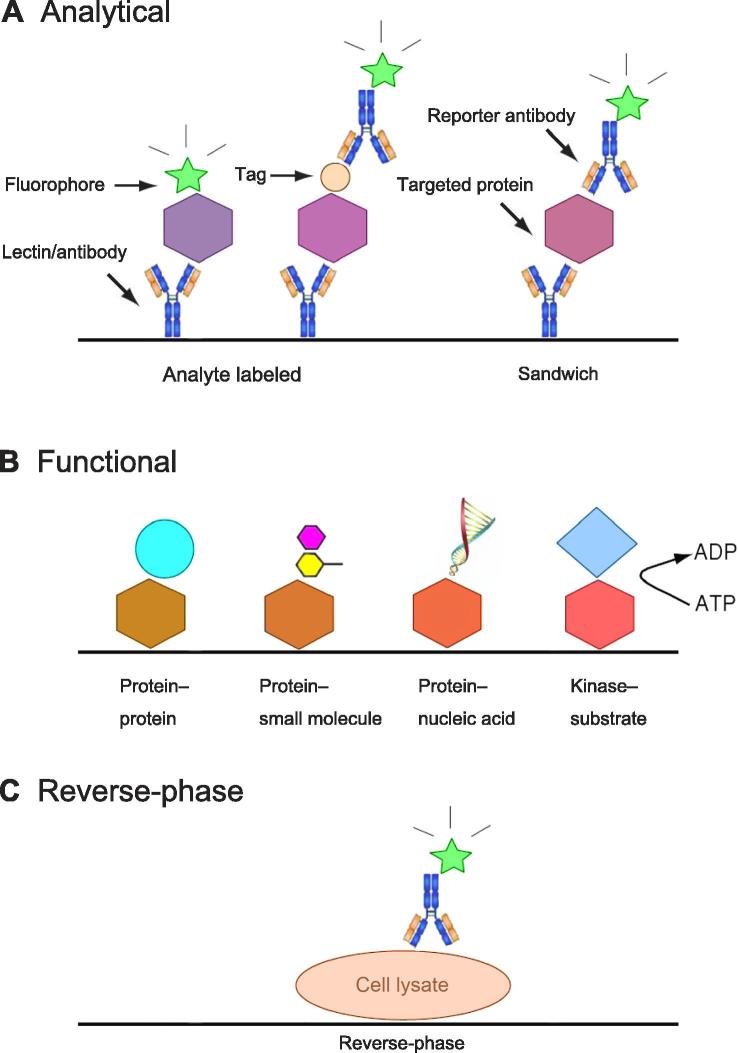
**Classification of three types of protein arrays** **A.** Analytical protein array is usually composed of well-characterized affinity reagents as immobilized probes, such as antibodies and lectins, to detect and/or quantify a large number of proteins present in a complex biological sample. In this class of arrays, targeted proteins can be detected either by direct labeling or using a reporter antibody in sandwich assay format. **B.** Functional protein arrays have broad applications in studying the biochemistry properties of proteins, such as protein binding activities and enzyme–substrate relationships. **C.** Reverse-phase protein arrays, comprised of many lysate samples, offer a platform to analyze signaling pathways.

In general, a protein array/microarray, also known as a protein chip, is formed by immobilizing individually-purified proteins onto a microscopic slide-based surface by using a robotic system, which can be either a contact [7] or a noncontact printing tool [8]. Alternatively, the proteins to be immobilized on an array can also be synthesized *in situ* [9]. On the basis of their fabrications and applications, protein arrays can be classified into three major categories: analytical, functional, and reverse-phase protein arrays (RPPAs) [6] (Figure 1). Analytical protein arrays, comprised of well-characterized affinity reagents, such as antibodies, lectins, and aptamers, are generally used to detect and/or quantify many proteins present in cultured cells and tissues [10], [11]. Thus, a wide variety of biological samples, such as lysates of cells, tissues, and tumors, can be readily assayed using this type of protein arrays, as evidenced by their wide uses in biomarker identification, protein expression profiling, clinical diagnosis/prognosis, as well as environmental and food safety analysis [12]. However, applications of this type of analytical protein arrays are limited by the availability and quality of the affinity reagents. Indeed, more and more researchers start to realize the importance of generating a large number of renewable antibodies that have been substantially characterized and validated for their binding specificity, affinity, and applications [13].

Functional protein arrays, which are usually constructed by spotting a large number of individually-purified recombinant proteins encoded by a given organism [6], [14], [15], represent another important class of protein arrays. When >60% of full length proteins encoded by a given organism are presented on a functional protein array, they can be referred as proteome arrays. To this date, proteome arrays have been constructed for several important model organisms, such as *Escherichia coli* [16], *Saccharomyces cerevisiae* [17], and humans [3], [18]. In recent years, proteins over-expressed in tissue culture or *in vitro* translated without further purification have also been used to fabricate functional protein arrays [19], [20]. In general, functional protein array-based approaches are useful to query various types of binding activities of proteins, such as protein–protein [7], [21], protein−peptide [22], protein–nucleic acids [23], [24], protein−glycan [25], protein–small molecules [26], and protein−lipid interactions [7]. Furthermore, they can also be used to investigate protein PTMs, such as protein phosphorylation [27], [28], ubiquitylation [29], acetylation [30], [31], and *S*-nitrosylation [32]. Applications of functional protein arrays have been dramatically expanded to translational research over the past decade. The most rapidly growing application of the functional protein arrays is, perhaps, in the field of serological biomarker identification. Such application has stemmed from the traditional serological studies, which search for the diagnostic identification of autoantibodies that are commonly found in serum samples of cancer patients. Although the exact molecular mechanisms underlying this phenomenon still remain unclear, it is generally speculated that a patient’s own aberrantly-expressed proteins in cancerous tissues could trigger an immune response [33]. Based on a similar principle, all of the proteins presented on a human proteome array can be assayed for their immunogenic activity via performing serum profiling assays, to identify serological biomarkers associated with a human disease or cancer [34].

RPPA, also known as a protein lysate array, is a special format of protein microarray. Instead of capturing analytes of a biological sample with affinity reagents, the analytes themselves, often cell or tissue lysates, as well as body fluids, in serial dilutions, are immobilized and arrayed on a glass slide at high density to form a RPPA [35]. Subsequently, antibodies with known binding properties are individually incubated onto the RPPA to examine specific epitopes, protein sequences, and/or structures. Since many lysate spots can be readily fit on a single glass slides, this technology offers a high-throughput platform for rapidly assaying a particular signaling event across many tissues or cell lines. Therefore, RPPA allows for examination of key signaling components of an intracellular pathway (or biomarker patterns) among many samples in a high-throughput fashion [36]. It has been used for studying differentially-regulated signaling networks in cell lines and clinical samples [37]. Therefore, such multifaceted and diverse spectra of various types of protein arrays have enabled their applications and potential uses in the cancer biomarker research (Table 1).

## Applications of analytical protein array for cancer biomarker discovery

The analytical protein array has been applied to identify biomarkers for a wide variety of cancers, because it allows simultaneously monitoring hundreds of thousands of protein samples in cells or tissues. One early and remarkable study was reported by Orchekowski et al. [38] in 2005. The authors used an antibody array, comprised of 92 antibodies, to profile protein contents in sera collected from 142 individuals with pancreatic cancer or benign pancreatic diseases, and from healthy individuals. As a result, they were able to identify protein signatures that could distinguish cancer patients from normal controls with a sensitivity and specificity of >90%. This study demonstrated the development and application of analytical arrays for identification of potential cancer biomarkers. In another study, Ingvarsson et al. [39] employed a special antibody array, comprised of various recombinant single-chain fragment-variable (scFv) antibodies, and profiled serum samples collected from pancreatic cancer patients to generate protein signatures of each sample. As negative controls, they also profile sera from healthy controls. Interestingly, they identified a protein signature consisting of 21 proteins that was potentially associated with cancer patients, who had a life expectancy of <12 months. In a more recent multicenter trial, analytical protein microarrays have also been applied for the biomarker discovery in pancreatic cancer [40]. This study aimed to identify a diagnostic, serological protein signature for pancreatic ductal adenocarcinoma (PDAC). Gerdtsson et al. [40] employed an antibody array, which consisted of 293 recombinant antibodies targeting immunoregulatory and cancer-associated antigens. They applied this array to a multicenter trial, comprised of a serum sample cohort of 338 patients with either PDAC or other pancreatic diseases (OPD) and controls with nonpancreatic conditions (NPC), to better mimic a real-life scenario. Consequently, they identified a multiplex biomarker panel of 10 serum proteins that could distinguish PDAC from controls with high sensitivity and specificity (91%–100%), demonstrating the potential of utilizing a multiplex biomarker panel-based immunoassay to improve PDAC diagnosis. All of the studies above represented the application of analytical protein arrays in the discovery of pancreatic cancer biomarkers, which may have the potential to achieve more accurate diagnosis and screening for high-risk individuals.

In addition to pancreatic cancer, breast cancer is another example that analytical protein arrays have been widely adopted as an unbiased screening tool for profiling the protein contents in cell lines, tissues, and patient sera. In an early attempt, Woodbury et al. [41] employed an antibody array to detect hepatocyte growth factor (HGF) as a biomarker in the sera collected from breast cancer patients using tyramide signal amplification (TSA). Another type of sample, namely the interstitial fluids collected from breast cancer patients, was also analyzed in a similar way. Using a variety of different methods, including an anti-cytokine antibody array, Celis et al. [42] analyzed the microenvironment of cells derived from breast cancer in search for potential biomarkers and therapeutic targets. In a follow-up study, the same research group [43] focused on the adipocytes, which also contribute to the tumor environment, using multiple methods including an antibody microarray. They were able to provide a landscape of the mammary fat proteomes that were relevant to high-risk breast cancer. Furthermore, Lin et al. [44] employed an anti-cytokine antibody array to detect the expression levels of different cytokines in breast cancer, resulting in the identification of interleukin 8 for its crucial role in breast cancer oncogenesis. Proteome-wide comparisons between the breast cancer and normal tissues have also been explored. For instance, Hudelist et al. [45] used a microarray composed of a commercial panel of 378 monoclonal antibodies to identify dysregulated protein expression levels within breast cancer cells as compared with those in normal tissues. As a result, some potential biomarkers, including casein kinase 1 epsilon (CSNK1E), p53 (TP53), cell division cycle 25C (CDC25C), annexin A11 (ANX11), eukaryotic translation initiation factor 4E (EIF4E) and mitogen-activated protein kinase 7 (MAPK7), were successfully identified to have increased expression levels in breast cancer.

In addition to the understanding of the biological mechanisms, analytical protein arrays have also been applied to profile drug resistance. In one study, Smith et al. [46] employed an array of 224 antibodies that could recognize various signaling components of diverse pathways, and determined the changes in the expression levels of these signaling components relevant to doxorubicin resistance. This study demonstrated the effectiveness of this tool in understanding the systematic effects of different therapeutic regimens, allowing for better understanding of the cause of drug resistance. Therefore, this study showed the potential in improving personalized medicine.

Plant and fungal lectins have long been known to specifically recognize simple or complex glycan structures on proteins or cell surfaces. Researchers have taken advantages of this special protein family as affinity reagents to understand the physiological role of glycosylation in various systems. For example, Kuno et al. [47] created an antibody-assisted lectin profiling assay to search for glycol-biomarkers. They used a model glycoprotein, podoplanin (hPod), to demonstrate that this pipeline could identify glycol-biomarker for metastasized glioblastoma cells. Because of this special binding property, lectins have also been employed recently as capture reagents for construction of analytical protein arrays. Indeed, two research groups pioneered the development and application of lectin arrays in profiling glycan structures of cell lysates and live cells [48], [49]. Tao et al. spotted 98 commercial lectins on nitrocellulose-coated glass slide and profiled accessible glycans of 24 live mammalian cell lines [49]. When they compared the glycan profiles obtained from a sphere cell population, which was enriched for cancer stem cells, and its parental cells (*e.g.*, MCF7 cells), three lectins, namely *Lycopersicon esculentum* lectin (LEL), *Aleuria aurantia* lectin (AAL), and wheat germ agglutinin (WGA), could preferentially capture MCF cells but not the sphere cells. To confirm whether these lectins could serve as biomarkers and enrich cancer stem cells, the authors used a mouse xenograft model and showed that LEL-depleted MCF cells were much more tumorigenic than the parental MCF cells. Later on, Huang et al. [50] spotted 37 commercially-available lectins, which could specifically recognize both *N*- and *O*-linked glycans, to form a lectin array, and applied it to obtain the lectin signatures of gastric cancer (GC) and gastric ulcer. They observed that glycosylation level was much higher in the paraffin-embedded GC tissues with a distinct lectin/glycan signature from that of gastric ulcer tissues. Two lectins, *Maclura pomifera* lectin (MPL) and *Vicia villosa* lectin (VVA), were further validated as biomarkers for GC via a lectin histochemistry assay. In another study, Nakajima et al. [51] profiled the lectin–glycan interactions via probing total protein preparations extracted from a large number of paraffin-embedded colorectal cancer and normal epithelium samples on a lectin array comprised of 45 lectins. In their validation studies, one lectin, *Agaricus bisporus* lectin (ABA), was found to show statistically significant association with recurrence of the curatively-resected colorectal cancer.

## Applications of functional protein array for cancer biomarker discovery

When a functional protein array is used for serum profiling, autoantibodies are usually detected as biomarkers for diagnosis of cancer appearance and for monitoring the cancer progress due to their stability, specificity, and ease of detection, as compared with other serological components [52]. Although the first proteome array consisting of 5800 unique yeast proteins spotted on a single glass slide was introduced in 2001 by Zhu et al. [7], functional protein arrays have become a popular tool for serum profiling only after the human proteome arrays composed of hundreds of thousands of individually-purified human proteins were constructed several years later [53], [54]. In general, the following approach is used for discovery and validation of serological biomarkers: first, each patient serum sample is diluted (*e.g.*, 1000-fold) in TBST and incubated on a pre-blocked human proteome microarray (*e.g.*, HuProt), followed by a stringent washing step. Next, captured human immunoglobulin on the human protein arrays is detected using fluorescently-labeled anti-human secondary antibodies (*e.g.*, anti-IgG, anti-IgM, or anti-IgA) for detection. Binding signals are then acquired with a microarray scanner and analyzed with GenePix software. Since most scanners have two laser sources, anti-IgG and anti-IgM can often be multiplexed. Unlike the traditional serological techniques, such as ELISA, agglutination, precipitation, and indirect immunofluorescence, which are all based on known antigens, functional protein array-based serum profiling offers an unbiased discovery tool for identification of novel biomarkers via surveying the entire human proteome in a single binding assay. It is also more sensitive than the traditional methodologies because each antigen protein on a functional protein array is purified with known identity. One representative study of such was reported in 2007 by Hudson et al. [55]. The authors applied a human functional protein array, composed of 5005 purified human proteins, to screen for autoantibody biomarkers in ovarian cancer. After screening 30 serum samples from cancer patients and 30 from healthy individuals, the authors identified 94 antigens that were preferentially recognized by sera in cancer patients. Four antigens, lamin A, lamin C, structure-specific recognition protein-1 (SSRP1), and Ral binding protein 1 (RALBP1), were further validated using immunoblotting analysis and tissue arrays.

An important goal of identifying cancer biomarkers is to define new strategies for early diagnosis that would allow early intervention with current therapies to improve patient survival rates. Moreover, since cancer-associated autoantibodies often target proteins that are mutated, modified, or aberrantly expressed in tumor cells, they could also be considered immunologic reporters that could uncover the etiology underlying tumorigenesis [56]. There is an urgent need to develop high-throughput diagnostic techniques for early diagnosis and treatment of prostate cancer (PCa). Adeola et al. [57] utilized an array of 123 tumor-associated antigens to measure the autoantibodies in serum samples from patients with PCa. They also used samples collected from patients with benign prostatic hyperplasia (BPH) and healthy control group as negative controls in South Africa. They were able to identified 41 potential markers for the diagnosis and treatment of PCa. The quantitative analysis showed that the antibody titers of G antigen 1 (GAGE1), rhophilin associated tail protein 1 (ROPN1), sperm protein associated with the nucleus, X-linked, family member A1 (SPANXA1), and protein kinase C zeta (PRKCZ) in the serum samples from PCa patients were higher than those from BPH patients, and melanoma antigen family B1 (MAGEB1) and PRKCZ were found to be highly expressed. Differential expression analysis showed that, as compared with BPH patients and healthy control subjects, expression of 24 different antigens in PCa patients was significantly up-regulated and expression of the 11 antigens was down-regulated. This study provides a valuable demonstration of the power of the functional protein arrays for the identification of autoantibodies, tumor-associated antigens, and applications for discovery of novel cancer biomarkers.

While the uniformity and high sensitivity of protein samples are the obvious advantages of the functional protein arrays, their applications can be limited by the extent of protein coverage. To this end, many research groups and commercial entities have begun to expand the availability of proteome-wide arrays. From a technological point of view, functional protein arrays of high reliability and quality are becoming more readily available (Table 2). Indeed, a functional protein array with the coverage of a given proteome would be the ideal tool for the discovery of novel cancer autoantibody biomarkers. For example, a functional human proteome array (*e.g.*, HuProt array vII), comprised of ∼17,000 full-length proteins with ∼75% coverage of the human proteome, was employed in a study by Yang et al. [3] to discover and validate serum autoantigens with potential for diagnosis and prognosis of GC. The authors assembled an impressive large set of serum samples (*N* = 1401) collected from 537 GC patients and 314 individuals of GC-related diseases, as well as 550 healthy controls. To ensure the success of this approach by avoiding potential overfitting problems, they adopted the two-phase strategy for biomarker identification [54]. In phase I, the HuProt arrays were surveyed with a smaller cohort of 87 serum samples from GC patients and healthy controls to screen for candidate autoantigens associated with GC. In phase II, a focused protein array of low cost was fabricated by spotting the identified candidate proteins in a 2 × 6 format on a single slide to allow for simultaneously profiling groups of 12 serum samples per slide. A much larger cohort of 914 samples were then assayed on these focused arrays in a double-blind fashion to validate the candidates identified in Phase I. Finally, the authors tested the performance of those validated biomarkers in the traditional ELISA assays, aiming at future clinical applications. As a result, four autoantigens, including constitutive photomorphogenesis protein (COP9) signalosome complex subunit 2 (COPS2), cathepsin F (CTSF), 5′-nucleotidase ecto (NT5E), and telomeric repeat binding factor 1 (TERF1), were confirmed as a new panel of biomarkers that could discriminate GC patients from healthy individuals with approximately 95% sensitivity and 92% specificity. Finally, the authors tested combinations of these individual markers and suggested that they could also serve as independent predictors of the overall survival rates of the GC patients.

In addition to the representative works discussed above, functional protein arrays have been used for hunting serological biomarkers in other cancers, such as lung cancer [58], colorectal cancer [59], breast cancer [60], and head and neck cancer [61], to name a few.

## Applications of RPPA for cancer biomarker discovery

The term “reverse-phase protein array” was proposed by Paweletz and colleagues in 2001 as opposed to antibody array [35]. Instead of spotting down affinity reagents to detect proteins-of-interest in a complex biological sample, lysates of cells or tissues are immobilized on the array surface to form a RPPA. While various kinds of antibody arrays have been applied to investigate breast cancers, RPPAs have also been used for the same purpose. Although analytical and functional protein arrays are the preferred tools to determine many different attributes for one analyte, RPPA offers an interesting platform that allows the researchers to focus on understanding a specific process among many different cell types or patient samples in parallel [62]. One obvious advantage of RPPA is to allow evaluation of the phosphorylation status of important signaling components of a given signaling pathway. For example, Rapkiewicz et al. [37] lysed archival cytological aspirate smears and frozen fine-needle aspirate samples to form a RPPA. Using commercial antibodies, they showed that proteins of interest could still be reliably quantitated even at low abundance and in either phosphorylated or un-phosphorylated forms.

Using RPPA as a tool, large panels of cancer cells have been studied and some potential biomarkers have been discovered. For example, Mendes et al. [63] fabricated a RPPA with lysates extracted from 90 different cell lines of 12 different cell types. Using phosphosite-specific antibodies targeting different signal transduction pathways, such as the phosphoinositide 3-kinase (PI3 K), epidermal growth factor receptor (EGFR), and vascular endothelial growth factor (VEGF) pathways, they were able to determine that the PI3K signaling pathway was up-regulated in different tumor types, and that the VEGF-angiogenesis pathway was down-regulated in hematopoietic cancers. Interestingly, they also observed that the EGFR signaling was the most heterogeneous pathway across all the cell lines tested, which provides important clinical implications for therapeutic target. In a recent study, Conti et al. [64] applied the RPPA technology to identify crosstalk between different signaling pathways using a set of 34 soft tissue sarcoma (STS) bone metastasis samples by comparing with those in healthy bone tissues. They identified that proteins associated with cellular matrix remodeling, cell adhesion, and growth/survival were elevated in bone metastasis than in normal bones. Furthermore, they found that linkage between syndecan-1, pY576/577-focal adhesion kinase (FAK), pY317-SH2 domain containing transforming protein (SHC) and EGFR, pY1135/1136-insulin-like growth factor (IGF), PI3K/AKT was a prominent feature of STS bone metastasis, while elevated linkage between receptor activator of nuclear factor kappa-B ligand (RANKL) and pT37/46-eukaryotic translation initiation factor 4E-binding protein 1 (4EBP1), EGFR, pY1135/1136- IGF-1 receptor (IGF1R), pY41-Src, pY317-SHC, PI3K p110 gamma (PI3Kp110γ) was associated with short survival. Their study provided clues to understand the linkage between cellular matrix remodeling and cell adhesion, suggesting that growth signaling might drive STS metastasis and examination of the phosphorylation status of these signaling molecules could be used for prognostic strategies.

A more exciting direction, perhaps, is to apply RPPA for the discovery of potential drug targets. For example, VanMeter et al. [65] used RPPAs to quantitatively detect EGFR phosphorylations in samples from patients of non-small-cell lung cancer (NSCLC) carrying mutant EGFR compared with those carrying wild type EGFR, and revealed simultaneously-elevated phosphorylation at Tyr-1148 and Tyr-1068 and reduced phosphorylation at Tyr-1045 of EGFR. In addition, they also detected reduced phosphorylation in signaling proteins related to EGFR including the human epidermal growth factor receptor 2 (HER2) at Tyr-1248, insulin receptor substrate 1 (IRS-1) at Ser-612, and SMAD at Ser-465/467. To assess which subset of phosphorylations was associated with ligand induction, they also evaluated the phosphorylation time course of 115 signaling proteins in NSCLC cell lines with mutant and wild-type EGFR after EGF ligand stimulation. Interestingly, following EGF ligand stimulation, EGFR mutant cell line H1975 with L858R showed phosphorylation at Tyr-1045 of EGFR and at Tyr-1248 of HER2 with a similar pattern to that found in tumor tissue. Additionally, persistence of phosphorylation for AKT at Ser-473 was found in EGFR mutant cell line H1975. Their studies explored multiple site-specific phosphoproteins *in vivo* in cell lines containing the EGFR tyrosine kinase domain mutations and provided the key insights into the potential drug targets for NSCLC.

## Outlook

**Figure 2:**
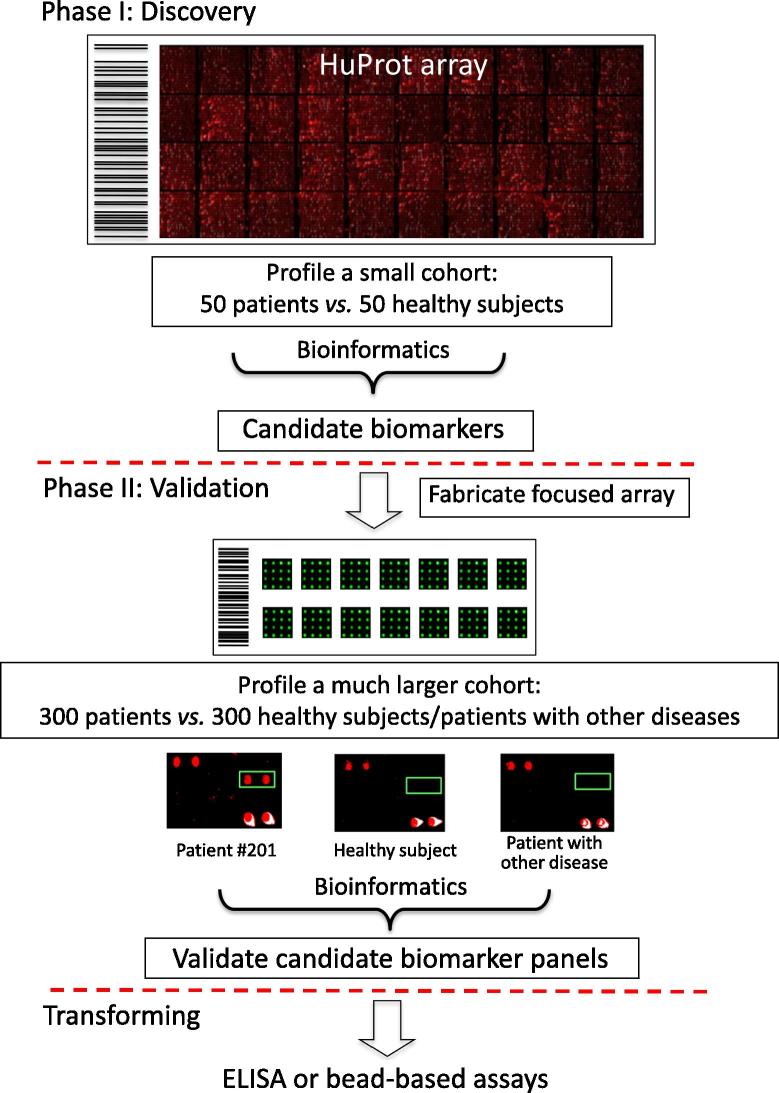
**Scheme of the two-phase strategy for biomarker identification using functional protein arrays** In Phase I, a small cohort is used to rapidly identify a group of candidate biomarkers via serum profiling assays on a HuProt array of high cost. Because a small number of arrays are needed, cost of the experiments is relatively low. In Phase II, a focused protein array of low cost is fabricated by spotting down candidate proteins identified in Phase I. A much larger cohort is assayed on the focused arrays in a double-blind fashion to validate the candidates identified in Phase I. Finally, the validated biomarkers are transformed into a clinically-friendly assay platform, such as ELISA. HuProt, Human Proteome Microarray; ELISA, enzyme-linked immunosorbent assay.

One of the most important goals for oncologists worldwide is to achieve early diagnosis and make accurate prognostic predictions. This would require a panel of biomarkers that, ideally, would be non-invasive and of high sensitivity and specificity. We believe that protein-based array approaches are playing and will continue to play a dominant role in cancer biomarker identification. This is because many cancer-relevant mutations, as well as aberrant expression, are protein-based and happen somatically. Therefore, it is no doubt that proteomics will provide the dominant driving force to achieve this ultimate goal for cancer researchers in this century. Protein array has been recognized as a robust tool in the field of clinical proteomics. With the tremendous growth in protein array-based methods and their popular uses that have been witnessed in recent studies, we believe that the protein array technology will become a powerful and popular tool for the discovery of novel biomarkers for cancer early diagnosis and prognosis. Functional protein arrays, in particular, are well poised to improve new personalized and novel targeted therapies. Ideally, a human protein array developed for such a purpose should need new strategies to overcome the current shortages: (1) the key issue for analytical and reverse phase protein arrays is getting both high-quality and sustainable antibodies to effectively control the cross-reactivity and (2) the key issue for functional protein array is its ability to cover the entire human proteome, to enable a comprehensive screen for the autoantigens. Meanwhile, due to the high cost of using high-throughput protein array, an effective strategy to overcome this obstacle is recommended, that is, to apply the two-phase strategy [54] (Figure 2). Indeed, most early studies in biomarker discovery failed to provide validation via testing additional cohorts and therefore, some of these potential biomarkers might be overfitting. Our two-phase strategy was designed to avoid potential overfitting problem by implementing a validation step in phase II [3], [54]. Another important issue with the current biomarker discovery is that translation to clinical applications has had only limited success, although numerous candidate biomarkers can be found in the literature. We believe that other than the overfitting issue as discussed above, there is a lack of transforming these biomarkers into a clinically-friendly assay system, such as ELISA-based detection methodology. Indeed, to remedy this problem, our team has tried to convert the functional protein array-based assays into ELISA-based tests [3], [54]. With all of these improvements, we believe that protein array technology will soon become a dominant tool for biomarker discovery in cancers.

## Competing interests

The authors declare no conflict of interest.

## Figures and Tables

**Table 1:** **Protein arrays for the cancer biomarker discovery**

**Types of array**	**Probes of array**	**Analytes**	**Applications**	**Pros**	**Cons**	**Refs.**
Analytical	Antibodies	Tissue/cell lysates, body fluids	Profiling protein expression	Low cost, easy to make, detecting multiple proteins	Relying on availability and quality of existing antibodies	[6]

	Lectins	Tissue/cell lysates, body fluids, live cells	Profiling glycosylations	Low cost, easy to make, profiling live cells	Relying on availability and quality of existing lectins	[11]

Functional	Purified or unpurified proteins	Body fluids	Profiling autoimmune reactions against transiently expressed proteins	Versatile applications, surveying an entire proteome unbiasedly	High cost, difficult to fabricate	[14], [15]

Reverse-phase	Fractionated lysates	Antibodies	Profiling dysregulated signaling networks	Low cost, easy to make, detecting multiple proteins	Relying on availability and quality of existing antibodies	[6]

**Table 2:** **Available high-content functional protein arrays**

**Types of array (species)**	**No. of proteins**	**Proteome coverage**	**Company/lab**	**Refs.**
HuProt (human)	20,000	>70%	CDI Laboratories, Zhu Lab	[3], [18]
ProtoArray (human)	>9000	∼45%	Life Technologies	[66]
PrESTs (human protein fragments)	>20,000	N/A	Uhlen Lab	[67]
*Arabidopsis thaliana* (plant)	17,400	50%	Dinesh-Kumar/Snyder Labs	[68]
*Saccharomyces cerevisiae* (fungus)	5800	∼85%	Zhu/Snyder Labs	[7], [30]
*Mycobacterium tuberculosis* (bacterium)	4262	>98%	BC-Bio, Tao Lab	[69]
*Escherichia coli* (bacterium)	4256	>98%	Zhu/Chen Labs	[70]
NAPPA (human)	∼4000	∼15%	Labaer Lab	[71]
Pathogenic antigens (bacteria)	200–4000	N/A	Antigen Discovery, Felgner Lab	[72]
Herpesvirus (virus)	350	N/A	Zhu/Hayward Labs	[73]
Influenza (virus)	127	N/A	Carter Lab	[74]

*Note*: HuProt, Human Proteome Microarray; NAPPA, Nucleic Acid-Programmable Protein Array; PrESTs, protein epitope signature tags.

## References

[1] Griffith J., Duncan R.C., Hulka B.S. (1989). Biochemical and biological markers: implications for epidemiologic studies. Arch Environ Health.

[2] Subramanyam M., Goyal J. (2016). Translational biomarkers: from discovery and development to clinical practice. Drug Discov Today Technol.

[3] Yang L., Wang J., Li J., Zhang H., Guo S., Yan M. (2016). Identification of serum biomarkers for gastric cancer diagnosis using a human proteome microarray. Mol Cell Proteomics.

[4] Perez-Rivas L.G., Jerez J.M., Fernandez-De S.C.E., de Luque V., Quero C., Pajares B. (2012). Serum protein levels following surgery in breast cancer patients: a protein microarray approach. Int J Oncol.

[5] Zhou J., Belov L., Solomon M.J., Chan C., Clarke S.J., Christopherson R.I. (2011). Colorectal cancer cell surface protein profiling using an antibody microarray and fluorescence multiplexing. J Vis Exp.

[6] Sutandy FX, Qian J, Chen CS, Zhu H. Overview of protein microarrays. Curr Protoc Protein Sci 2013;Chapter 27: Unit 27.1. 10.1002/0471140864.ps2701s72 PMC3680110 23546620

[7] Zhu H., Bilgin M., Bangham R., Hall D., Casamayor A., Bertone P. (2001). Global analysis of protein activities using proteome chips. Science.

[8] Delehanty J.B., Ligler F.S. (2003). Method for printing functional protein microarrays. Biotechniques.

[9] Festa F., Rollins S.M., Vattem K., Hathaway M., Lorenz P., Mendoza E.A. (2013). Robust microarray production of freshly expressed proteins in a human milieu. Proteomics Clin.

[10] Haab BB, Dunham MJ, Brown PO. Protein microarrays for highly parallel detection and quantitation of specific proteins and antibodies in complex solutions. Genome Biol 2001;2:RESEARCH0004. 10.1186/gb-2001-2-2-research0004 PMC25774 11182887

[11] Syed P., Gidwani K., Kekki H., Leivo J., Pettersson K., Lamminmäki U. (2016). Role of lectin microarrays in cancer diagnosis. Proteomics.

[12] Kumble K.D. (2003). Protein microarrays: new tools for pharmaceutical development. Anal Bioanal Chem.

[13] Uhlen M., Bandrowski A., Carr S., Edwards A., Ellenberg J., Lundberg E. (2016). A proposal for validation of antibodies. Nat Methods.

[14] Zhu H., Cox E., Qian J. (2012). Functional protein microarray as molecular decathlete: a versatile player in clinical proteomics. Proteomics Clin Appl.

[15] Moore C.D., Ajala O.Z., Zhu H. (2016). Applications in high-content functional protein microarrays. Curr Opin Chem Biol.

[16] Liu C.X., Wu F.L., Jiang H.W., He X., Guo S.J., Tao S.C. (2014). Global identification of CobB interactors by an *Escherichia coli* proteome microarray. Acta Biochim Biophys Sin (Shanghai).

[17] Gelperin D.M., White M.A., Wilkinson M.L., Kon Y., Kung L.A., Wise K.J. (2005). Biochemical and genetic analysis of the yeast proteome with a movable ORF collection. Genes Dev.

[18] Hu C.J., Song G., Huang W., Liu G.Z., Deng C.W., Zeng H.P. (2012). Identification of new autoantigens for primary biliary cirrhosis using human proteome microarrays. Mol Cell Proteomics.

[19] Ma D., Baruch D., Shu Y., Yuan K., Sun Z., Ma K. (2012). Using protein microarray technology to screen anti-ERCC1 monoclonal antibodies for specificity and applications in pathology. BMC Biotechnol.

[20] Eyles J.E., Unal B., Hartley M.G., Newstead S.L., Flick-Smith H., Prior J.L. (2007). Immunodominant *Francisella tularensis* antigens identified using proteome microarray. Proteomics.

[21] Popescu S.C., Popescu G.V., Bachan S., Zhang Z., Seay M., Gerstein M. (2007). Differential binding of calmodulin-related proteins to their targets revealed through high-density Arabidopsis protein microarrays. Proc Natl Acad Sci U S A.

[22] Jones R.B., Gordus A., Krall J.A., MacBeath G. (2006). A quantitative protein interaction network for the ErbB receptors using protein microarrays. Nature.

[23] Zhu J., Gopinath K., Murali A., Yi G., Hayward S.D., Zhu H. (2007). RNA-binding proteins that inhibit RNA virus infection. Proc Natl Acad Sci U S A.

[24] Hu S., Xie Z., Onishi A., Yu X., Jiang L., Lin J. (2009). Profiling the human protein-DNA interactome reveals ERK2 as a transcriptional repressor of interferon signaling. Cell.

[25] Kung L.A., Tao S.C., Qian J., Smith M.G., Snyder M., Zhu H. (2009). Global analysis of the glycoproteome in *Saccharomyces cerevisiae* reveals new roles for protein glycosylation in eukaryotes. Mol Syst Biol.

[26] Huang J., Zhu H., Haggarty S.J., Spring D.R., Hwang H., Jin F. (2004). Finding new components of the target of rapamycin (TOR) signaling network through chemical genetics and proteome chips. Proc Natl Acad Sci U S A.

[27] Zhu H., Klemic J.F., Chang S., Bertone P., Casamayor A., Klemic K.G. (2000). Analysis of yeast protein kinases using protein chips. Nat Genet.

[28] Ptacek J., Devgan G., Michaud G., Zhu H., Zhu X., Fasolo J. (2005). Global analysis of protein phosphorylation in yeast. Nature.

[29] Lu J.Y., Lin Y.Y., Qian J., Tao S.C., Zhu J., Pickart C. (2008). Functional dissection of a HECT ubiquitin E3 ligase. Mol Cell Proteomics.

[30] Lin Y.Y., Lu J.Y., Zhang J., Walter W., Dang W., Wan J. (2009). Protein acetylation microarray reveals that NuA4 controls key metabolic target regulating gluconeogenesis. Cell.

[31] Lu J.Y., Lin Y.Y., Sheu J.C., Wu J.T., Lee F.J., Chen Y. (2011). Acetylation of yeast AMPK controls intrinsic aging independently of caloric restriction. Cell.

[32] Foster M.W., Forrester M.T., Stamler J.S. (2009). A protein microarray-based analysis of *S*-nitrosylation. Proc Natl Acad Sci U S A.

[33] Tan E.M. (2001). Autoantibodies as reporters identifying aberrant cellular mechanisms in tumorigenesis. J Clin Invest.

[34] Benvenuto M., Mattera R., Masuelli L., Tresoldi I., Giganti M.G., Frajese G.V. (2017). The crossroads between cancer immunity and autoimmunity: antibodies to self antigens. Front Biosci (Landmark Ed).

[35] Paweletz C.P., Charboneau L., Bichsel V.E., Simone N.L., Chen T., Gillespie J.W. (2001). Reverse phase protein microarrays which capture disease progression show activation of pro-survival pathways at the cancer invasion front. Oncogene.

[36] Nishizuka S.S., Mills G.B. (2016). New era of integrated cancer biomarker discovery using reverse-phase protein arrays. Drug Metab Pharmacokinet.

[37] Rapkiewicz A., Espina V., Zujewski J.A., Lebowitz P.F., Filie A., Wulfkuhle J. (2007). The needle in the haystack: application of breast fine-needle aspirate samples to quantitative protein microarray technology. Cancer.

[38] Orchekowski R., Hamelinck D., Li L., Gliwa E., vanBrocklin M., Marrero J.A. (2005). Antibody microarray profiling reveals individual and combined serum proteins associated with pancreatic cancer. Cancer Res.

[39] Ingvarsson J., Wingren C., Carlsson A., Ellmark P., Wahren B., Engström G. (2008). Detection of pancreatic cancer using antibody microarray-based serum protein profiling. Proteomics.

[40] Gerdtsson A.S., Malats N., Säll A., Real F.X., Porta M., Skoog P. (2015). A multicenter trial defining a serum protein signature associated with pancreatic ductal adenocarcinoma. Int J Proteomics.

[41] Woodbury R.L., Varnum S.M., Zangar R.C. (2002). Elevated HGF levels in sera from breast cancer patients detected using a protein microarray ELISA. J Proteome Res.

[42] Celis J.E., Gromov P., Cabezon T., Moreira J.M., Ambartsumian N., Sandelin K. (2004). Proteomic characterization of the interstitial fluid perfusing the breast tumor microenvironment: a novel resource for biomarker and therapeutic target discovery. Mol Cell Proteomics.

[43] Celis J.E., Moreira J.M., Cabezon T., GromovP Friis E., Rank F. (2005). Identification of extracellular and intracellular signaling components of the mammary adipose tissue and its interstitial fluid in high risk breast cancer patients: toward dissecting the molecular circuitry of epithelial-adipocyte stromal cell interactions. Mol Cell Proteomics.

[44] Lin Y., Huang R., Chen L., Li S., Shi Q., Jordan C. (2004). Identification of interleukin-8 as estrogen receptor-regulated factor involved in breast cancer invasion and angiogenesis by protein arrays. Int J Cancer.

[45] Hudelist G., Pacher-Zavisin M., Singer C.F., Holper T., Kubista E., Schreiber M. (2004). Use of high throughput protein array for profiling of differentially expressed proteins in normal and malignant breast tissue. Breast Cancer Res Treat.

[46] Smith L., Watson M.B., O’Kane S.L., Drew P.J., Cawkwell L. (2006). The analysis of doxorubicin resistance in human breast cancer cells using antibody microarrays. Mol Cancer Ther.

[47] Kuno A., Kato Y., Matsuda A., Kaneko M.K., Ito H., Amano K. (2009). Focused differential glycan analysis with the platform antibody-assisted lectin profiling for glycan-related biomarker verification. Mol Cell Proteomics.

[48] Hsu K.L., Pilobello K.T., Mahal L.K. (2006). Analyzing the dynamic bacterial glycome with a lectin microarray approach. Nat Chem Biol.

[49] Tao S.C., Li Y., Zhou J., Qian J., Schnaar R.L., Zhang Y. (2008). Lectin microarrays identify cell-specific and functionally significant cell surface glycan markers. Glycobiology.

[50] Huang W.L., Li Y.G., Lv Y.C., Guan X.H., Ji H.F., Chi B.R. (2014). Use of lectin microarray to differentiate gastric cancer from gastric ulcer. World J Gastroenterol.

[51] Nakajima K., Inomata M., Iha H., Hiratsuka T., Etoh T., Shiraishi N. (2015). Establishment of new predictive markers for distant recurrence of colorectal cancer using lectin microarray analysis. Cancer Med.

[52] Heo C.K., Bahk Y.Y., Cho E.W. (2012). Tumor-associated autoantibodies as diagnostic and prognostic biomarkers. BMB Rep.

[53] Hu S., Xie Z., OnishiA Yu.X., Jiang L., Lin J. (2009). Profiling the human protein-DNA interactome reveals ERK2 as a transcriptional repressor of interferon signaling. Cell.

[54] Song Q., Liu G., Hu S., Zhang Y., Tao Y., Han Y. (2010). Novel autoimmune hepatitis-specific autoantigens identified using protein microarray technology. J Proteome Res.

[55] Hudson M.E., Pozdnyakova I., Haines K., Mor G., Snyder M. (2007). Identification of differentially expressed proteins in ovarian cancer using high-density protein microarrays. Proc Natl Acad Sci U S A.

[56] Orenes-Piñero E., Barderas R., Rico D., Casal J.I., Gonzalez-Pisano D., Navajo J. (2010). Serum and tissue profiling in bladder cancer combining protein and tissue arrays. J Proteome Res.

[57] Adeola H.A., Smith M., Kaestner L., Blackburn J.M., Zerbini L.F. (2016). Novel potential serological prostate cancer biomarkers using CT100+ cancer antigen microarray platform in a multi-cultural South African cohort. Oncotarget.

[58] Zhong L., Hidalgo G.E., Stromberg A.J., Khattar N.H., Jett J.R., Hirschowitz E.A. (2005). Using protein microarray as a diagnostic assay for non-small cell lung cancer. Am J Respir Crit Care Med.

[59] Babel I., Barderas R., Díaz-Uriarte R., Martínez-Torrecuadrada J.L., Sánchez-Carbayo M., Casal J.I. (2009). Identification of tumor-associated autoantigens for the diagnosis of colorectal cancer in serum using high density protein microarrays. Mol Cell Proteomics.

[60] Anderson K.S., Sibani S., Wallstrom G., Qiu J., Mendoza E.A., Raphael J. (2011). Protein microarray signature of autoantibody biomarkers for the early detection of breast cancer. J Proteome Res.

[61] Lin H.S., Talwar H.S., Tarca A.L., Ionan A., Chatterjee M., Ye B. (2007). Autoantibody approach for serum-based detection of head and neck cancer. Cancer Epidemiol Biomarkers Prev.

[62] Akbani R., Becker K.F., Carragher N., Goldstein T., de Koning L., Korf U. (2014). Realizing the promise of reverse phase protein arrays for clinical, translational, and basic research: a workshop report: the RPPA (Reverse Phase Protein Array) society. Mol Cell Proteomics.

[63] Mendes K.N., Nicorici D., Cogdell D., Tabus I., Yli-Harja O., Guerra R. (2007). Analysis of signaling pathways in 90 cancer cell lines by protein lysate array. J Proteome Res.

[64] Conti A., Espina V., Chiechi A., Magagnoli G., Novello C., Pazzaglia L. (2014). Mapping protein signal pathway interaction in sarcoma bone metastasis: linkage between rank, metalloproteinases turnover and growth factor signaling pathways. Clin Exp Metastasis.

[65] VanMeter A.J., Rodriguez A.S., Bowman E.D., Jen J., Harris C.C., Deng J. (2008). Laser capture microdissection and protein microarray analysis of human non-small cell lung cancer: differential epidermal growth factor receptor (EGPR) phosphorylation events associated with mutated EGFR compared with wild type. Mol Cell Proteomics.

[66] Stafford P., Halperin R., Legutki J.B., Magee D.M., Galgiani J., Johnston S.A. (2012). Physical characterization of the “immunosignaturing effect”. Mol Cell Proteomics.

[67] Sjöberg R., Sundberg M., Gundberg A., Sivertsson A., Schwenk J.M., Uhlén M. (2012). Validation of affinity reagents using antigen microarrays. N Biotechnol.

[68] Manohar M., Tian M., Moreau M., Park S.W., Choi H.W., Fei Z. (2015). Identification of multiple salicylic acid-binding proteins using two high throughput screens. Front Plant Sci.

[69] Deng J., Bi L., Zhou L., Guo S.J., Fleming J., Jiang H.W. (2014). *Mycobacterium tuberculosis* proteome microarray for global studies of protein function and immunogenicity. Cell Rep.

[70] Chen C.S., Korobkova E., Chen H., Zhu J., Jian X., Tao S.C. (2008). A proteome chip approach reveals new DNA damage recognition activities in *Escherichia coli*. Nat Methods.

[71] Miersch S., Bian X., Wallstrom G., Sibani S., Logvinenko T., Wasserfall C.H. (2013). Serological autoantibody profiling of type 1 diabetes by protein arrays. J Proteomics.

[72] Liang L., Felgner P.L. (2015). A systems biology approach for diagnostic and vaccine antigen discovery in tropical infectious diseases. Curr Opin Infect Dis.

[73] Zhu J., Liao G., Shan L., Zhang J., Chen M.R., Hayward G.S. (2009). Protein array identification of substrates of the Epstein-Barr virus protein kinase BGLF4. J Virol.

[74] Desbien A.L., Van Hoeven N., Reed S.J., Casey A.C., Laurance J.D., Baldwin S.L. (2013). Development of a high density hemagglutinin protein microarray to determine the breadth of influenza antibody responses. Biotechniques.

